# Exotic fishes that are phylogenetically close but functionally distant to native fishes are more likely to establish

**DOI:** 10.1111/gcb.16360

**Published:** 2022-08-04

**Authors:** Meng Xu, Shao‐peng Li, Jaimie T. A. Dick, Dangen Gu, Miao Fang, Yexin Yang, Yinchang Hu, Xidong Mu

**Affiliations:** ^1^ Pearl River Fisheries Research Institute Chinese Academy of Fishery Sciences Guangzhou China; ^2^ Key Laboratory of Prevention and Control for Aquatic Invasive Alien Species Ministry of Agriculture and Rural Affairs Guangzhou China; ^3^ Key Laboratory of Alien Species and Ecological Security (CAFS) Pearl River Fisheries Research Institute, Chinese Academy of Fishery Sciences Guangzhou China; ^4^ Zhejiang Tiantong Forest Ecosystem National Observation and Research Station, School of Ecological and Environmental Sciences East China Normal University Shanghai China; ^5^ Institute for Global Food Security, School of Biological Sciences Queen's University Belfast Belfast UK

**Keywords:** biological invasions, competition, Darwin’s naturalization hypothesis, ecological similarity, environmental filtering, exotic species, functional traits, phylogenetic distance

## Abstract

Since Darwin's time, degree of ecological similarity between exotic and native species has been assumed to affect the establishment success or failure of exotic species. However, a direct test of the effect of exotic–native similarity on establishment of exotics is scarce because of the difficulty in recognizing failures of species to establish in the field. Here, using a database on the establishment success and failure of exotic fish species introduced into 673 freshwater lakes, we evaluate the effect of similarity on the establishment of exotic fishes by combining phylogenetic and functional information. We illustrate that, relative to other biotic and abiotic factors, exotic–native phylogenetic and functional similarities were the most important correlates of exotic fish establishment. While phylogenetic similarity between exotic and resident fish species promoted successful establishment, functional similarity led to failure of exotics to become established. Those exotic species phylogenetically close to, but functionally distant from, native fishes were most likely to establish successfully. Our findings provide a perspective to reconcile Darwin's naturalization conundrum and suggest that, while phylogenetic relatedness allows exotic fish species to pre‐adapt better to novel environments, they need to possess distinct functional traits to reduce competition with resident native fish species.

## Graphical abstract

Using the data on the establishment success and failure of exotic fish species introduced into 673 freshwater lakes, we examined the factors driving establishment of exotic fishes. We illustrate that exotic‐native phylogenetic and functional similarities were the most important correlates of successful exotic fish establishment. Phylogenetic similarity between exotic and resident fishes promoted successful establishment, but functional similarity led to failure of exotics to become established. Our findings suggest that, while phylogenetic relatedness allows exotic fish species to pre‐adapt better to novel environments, they need to possess distinct functional traits to reduce competition with resident native fish species.

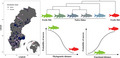

## 1 INTRODUCTION

The introduction and establishment of exotic species is occurring ever‐more frequently worldwide (Dawson et al., 2017; Pyšek et al., 2020; Seebens et al., 2021), and successful invaders have brought about widespread ecological, economic, and social consequences (Cuthbert et al., 2021; Pyšek & Richardson, 2010). Understanding and predicting which exotic species can invade successfully have become a major goal in ecology and conservation (Fristoe et al., 2021; Kolar & Lodge, 2001; Seebens et al., 2018). Among the myriad hypotheses to explain successful invasion (Enders et al., 2020), two hypotheses proposed by Darwin that focus on phylogenetic relatedness between exotic species and recipient communities have attracted much attention during the last two decades (Cadotte et al., 2018; Diez et al., 2008; Thuiller et al., 2010). On the one hand, Darwin posited that exotic species closely related to resident species would be more likely to establish successfully, because they might share similar adaptations to the local physical environment with their native relatives, which was referred to as the “pre‐adaptation hypothesis” (PAH) (Ricciardi & Mottiar, 2006). On the other hand, Darwin also stated that exotic species phylogenetically distinct from the native species would tend to be more successful, as they might suffer less competition and share fewer natural enemies with the native species, which was known as “Darwin's naturalization hypothesis” (DNH) (Daehler, 2001). These two opposing hypotheses have been referred to as “Darwin's naturalization conundrum” (Cadotte et al., 2018; Diez et al., 2008).

Many studies have investigated this conundrum across different taxonomic groups and ecosystems using observational approaches (Bezeng et al., 2015; Duncan & Williams, 2002; Li, Cadotte, et al., 2015; Park et al., 2020; Pinto‐Ledezma et al., 2020; Qian et al., 2021; Strauss et al., 2006). However, the vast majority of these studies were based primarily on observations of successfully invaded exotic species, but did not consider those that were introduced but failed to establish. These studies generally used the null model approach and considered exotic–native phylogenetic relatedness more distant than expected by chance as evidence that supports the DNH, with the opposite pattern supporting the PAH (Li, Cadotte, et al., 2015; Procheş et al., 2008). However, co‐occurrence of exotics with distantly related native species could arise either from exclusion of exotics by closely related native species or from elimination of native species by closely related exotics (Cadotte et al., 2018; Thuiller et al., 2010). For example, Li, Cadotte, et al. (2015) showed that closely related exotic species were more likely to establish (consistent with the PAH), but their strong competitive ability excluded their closely related native species, resulting in an apparent coexistence between distantly related exotic and native species (apparently consistent with the DNH) (Li, Cadotte, et al., 2015). Alternatively, besides pre‐adaptation to novel environments, competitive exclusion could also result in co‐occurrence of exotics with closely related native species (Mayfield & Levine, 2010). That is, even distantly related exotic species are more likely to establish (consistent with the DNH), they may then exclude distantly related native species that have low competitive ability (Mayfield & Levine, 2010), leading to an observed exotic–native phylogenetic clustering (apparently consistent with the PAH). Therefore, while offering important insights, it may be difficult to draw definitive conclusions about Darwin's naturalization conundrum based upon the *posterior* observation of successful invaders. By contrast, by considering both successful and failed exotic species in the field, we can examine directly how and to what extent phylogenetic distance affects the likelihood of establishment in nature. Positive effects of phylogenetic distance would indicate that distantly related exotics are more likely to establish, supporting the DNH, while negative effects would support the PAH. Further, we can include other biotic and abiotic factors conveniently into analyses and account for their potential confounding effects statistically. This approach is technically similar to several experimental studies (Feng et al., 2019; Jiang et al., 2010), but can reveal the effect of phylogenetic similarity on successful or failed establishment in complex real‐world ecosystems much more clearly and convincingly.

Based upon the assumption that closely related species should share similar traits (Cavender‐Bares et al., 2009; Webb et al., 2002), functional similarity has been assumed to play an important role similar to phylogenetic relatedness in predicting successful or failed invasions (Divíšek et al., 2018; Gallien & Carboni, 2017; Rocha & Cianciaruso, 2021; Yannelli et al., 2017). However, an increasing number of studies have recognized that functional and phylogenetic similarities may provide different, and perhaps complementary, information in capturing species differences (Cadotte et al., 2019; de Bello et al., 2017; Mazel et al., 2018). Phylogenetic relatedness may represent an integrative measure of functional similarity and capture some potentially important functions, which may not be covered by a limited number of measured traits (Cadotte et al., 2013). Several observational studies have compared the phylogenetic and functional similarity of exotic species to co‐occurring natives, and provided important insights into understanding their differential influences on invasion success (Marx et al., 2016; Ordonez, 2014; Pinto‐Ledezma et al., 2020). However, while phylogenetic and functional similarities may operate at the same time, these separate examinations make it difficult to reveal their synergistic effect. Considering phylogenetic and functional similarities simultaneously would help distinguish their magnitudes and directions in predicting the success or failure of exotic species, and may offer more insights into interpreting Darwin's naturalization conundrum.

In addition to exotic–native ecological similarities, multiple abiotic and biotic characteristics of the recipient communities may also affect the establishment of exotic species (Catford et al., 2009). For example, with increasing diversity of native species, more niche space is occupied, and the native community is also more likely to include species with strong competitive abilities. Therefore, native diversity may resist successful invasion (Beaury et al., 2020). High elevation and latitude and associated harsh environments may also limit establishment of exotics (Pearson et al., 2018). These multiple determinants of successful or failed invasion may make it difficult to identify the actual role of ecological similarity. Few studies to date have investigated the relative importance of ecological similarity compared with these confounding factors explicitly and identified their direct and indirect effects on the establishment of exotic species.

Here, using a unique database of 965 successful or failed establishments of introduced fishes in 673 freshwater lakes, we tested Darwin's naturalization conundrum by integrating phylogenetic and functional information. We also evaluated the relative importance of ecological similarity compared with biotic (native community taxonomic, phylogenetic, and functional diversity) and abiotic factors (geographical locations and water temperature). We revealed that, relative to native diversity, geographical, and environmental factors, ecological similarity was the most important factor that influenced the establishment of exotic fishes. However, the phylogenetic and functional similarities have opposing effects: phylogenetic similarity between exotic and resident species promoted successful establishment, but functional similarity led to failure of exotics to become established. Our findings provide a perspective to reconcile Darwin's naturalization conundrum and suggest that a successful invader should be phylogenetically closely related to natives but have distinct functional traits.

## 2 MATERIALS AND METHODS

### 2.1 Data collection

**FIGURE 1:**
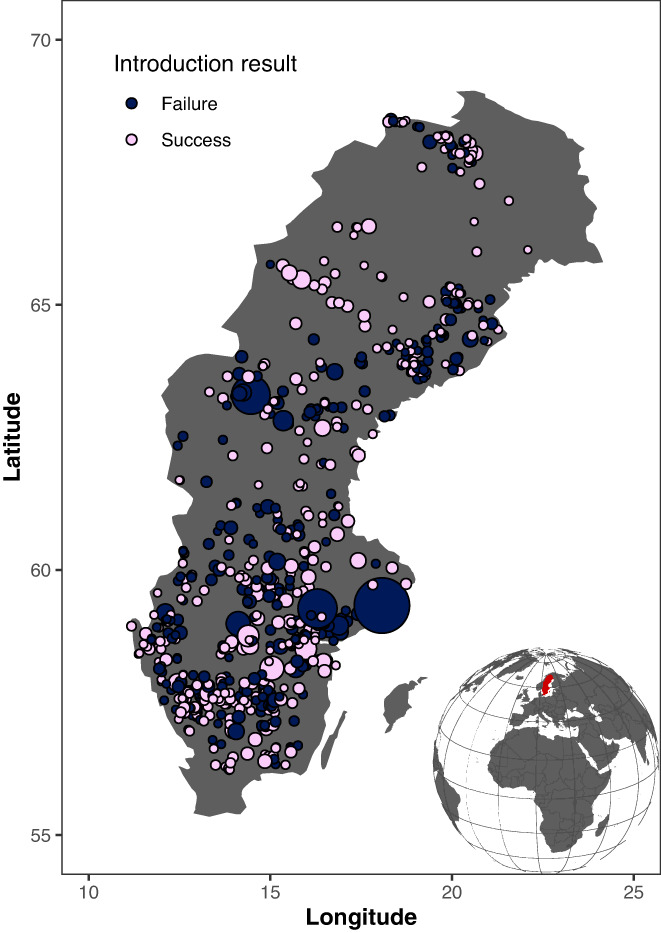
Geographical distribution and results of fish introductions into 673 freshwater lakes in Sweden. Circle size is proportional to lake area. Map lines delineate study areas and do not necessarily depict accepted national boundaries.

We acquired fish introduction data from a published dataset (Henriksson, Rydberg, & Englund, 2016) that includes 1157 intentional introductions that record the successful and failed establishment of 26 freshwater fish species introduced into 820 Swedish lakes from 1685 to 2002. Successful establishment means that the introduced fish species were present in the lake for ≥20 years, or that reproduction was observed earlier than that (Henriksson, Rydberg, & Englund, 2016). Most of these introductions were conducted for consumption or recreational fisheries and the outcome of introductions was often carefully monitored. Therefore, this dataset may have the best documentation for failures as well as successes of exotic fishes and is thus most appropriate for exploring our questions. For each lake, native species richness, lake area, elevation, longitude, and latitude were recorded. Accumulated and maximum water temperature were also calculated with the following approaches: accumulated water temperature was firstly calculated for 198 lakes using the available temperature readings; then, the accumulated water temperature was regressed on lake depth, lake area, and accumulated air temperature (modeling using latitude and elevation as predictors), obtaining the parameterized regression function; finally, using the regression function, the accumulated water temperature for all other lakes was estimated based on the known abiotic factors. Similar procedures were also conducted to calculate the maximum water temperature. We filtered the data as follows: (1) we omitted 73 lakes because the native species richness was recorded as 0 and thus the similarity between the introduced and native species could not be determined; (2) we omitted nine introductions of two hybrid species (*Salvelinus fontinalis* × *S. alpinus*; *S. namaycush* × *S. fontinalis*) because their genetic and functional information was unavailable; (3) we omitted one introduction in which the introduced species name in the dataset had been lost (Introduction_no. 512 in the original dataset); (4) we omitted three native species because only family names (Cottidae, Cyprinidae, and Gasteriodae) were recorded and thus genetic and functional information was unavailable for them; and (5) we inferred conservatively that one native species was absent in a specific lake when the authors were unable to assure its presence (the authors defined the uncertain presence as 0.5 in the original dataset, and we defined it as 0 in this study). Accordingly, we used a clean dataset that included 965 intentional introductions of 23 freshwater fish species into 673 lakes in Sweden (Figure 1). The maximum, median, and minimum native fish species numbers for these lakes are 27, 6, and 2, respectively. A total of 38 introduced and native fish species were involved in this study. We constructed the phylogenetic and functional distance matrices for all these introduced and native species to explore the effect of phylogenetic and functional similarities on the establishment of the introduced fishes.

### 2.2 Molecular phylogeny reconstruction

**FIGURE 2:**
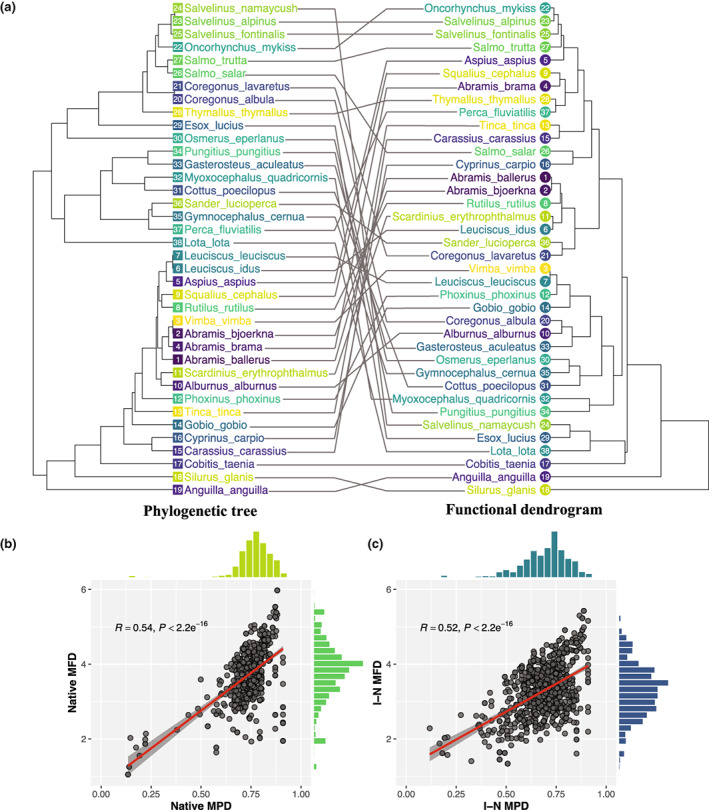
The relationships between the phylogenetic tree and functional dendrogram (a), between native phylogenetic and functional diversity (b), and exotic–native phylogenetic and functional similarities (c). The predictive lines (and 95% confidence bands) are derived from simple linear regressions. The distributions of these four indices are shown as marginal histograms. MPD denotes the mean phylogenetic distance of native species, while MFD denotes the mean functional distance. I‐N MPD denotes the mean phylogenetic distance between introduced exotic species and native species, while the I‐N MFD denotes the mean functional distance between introduced exotic species and native species.

We constructed the Bayesian phylogenetic tree for these species based upon three mitochondrial gene sequences (*cytb*, *COI*, and *16S rRNA*) obtained from GenBank and used *Carcharodon carcharias* (a fish species belonging to the Chondrichthyes) to serve as an outgroup. Sequences were aligned independently using MUSCLE v. 3.8.31(Edgar, 2004) and the best‐fit model of nucleotide substitution for each region was selected using jModelTest v. 2.1.10 (Posada, 2008). For each region, the “GTR + G + I” substitution model was selected and its parameter estimates were used in BEAUti (Remco et al., 2014), with an uncorrelated lognormal relaxed molecular clock model and a Yule speciation tree prior. The Bayesian phylogeny was reconstructed using BEAST v. 2.4.7 (Remco et al., 2014). The Bayesian MCMC chain was run for 10 million generations and convergence was checked using Tracer version v. 1.6.0 (http://beast.bio.ed.ac.uk/Tracer). The maximum clade credibility tree from the posterior distribution was used to quantify phylogenetic patterns with TreeAnnotator v. 2.4.7 (Remco et al., 2014). The reconstructed phylogeny represented the evolutionary relationship among introduced and resident fish species in all 673 Swedish freshwater lakes (Figure 2a). Based upon the phylogeny, we calculated the pairwise phylogenetic distance among all species using the *cophenetic.phylo* function of the *ape* R package (Emmanuel et al., 2004).

### 2.3 Functional dendrogram construction

We extracted 10 morphological traits of all 38 introduced and native fish species from the FISHMORPH database (Brosse et al., 2021). To our knowledge, this dataset represents the most comprehensive and best available data on fish traits to date, and it has been used in multiple studies to analyze the functional diversity of fish species (Su et al., 2022; Toussaint et al., 2018). The traits we considered include maximum body length, body elongation, vertical eye position, relative eye size, oral gape position, relative maxillary length, lateral body shape, pectoral fin vertical position, pectoral fin size, and caudal peduncle throttling. The methods used to measure these continuous traits and their associations with fish functions have been documented in Brosse et al. (2021). While these traits undoubtedly do not account for the entire range of fish functions, they provide key information on food acquisition, locomotion, nutrient cycling, and defense against predation (Villéger et al., 2017). We used the first five principal components (PC1‐PC5) of the ten morphological traits to calculate pairwise functional distances between introduced and native fish species, using the *dist* function of the *stats* package following a common analytical approach (Swenson, 2014). Based upon the distance matrix obtained, we constructed the functional dendrogram of all fish species using hierarchical clustering with the *hclust* function of the *stats* package and compared it to the phylogenetic tree generated above (Figure 2a). Alternatively, some physiological and reproductive traits may be directly obtained from online database such as FishBase (www.fishbase.se), which could also be used to calculate functional distances. However, much information on these traits was only qualitative or not available for the species in our study. While the Gower distance apparently can be applied to deal with categorical and missing data (Gower, 1971), integrating a mass of qualitative or missing trait values into our analysis would make the trait distance less clear and definitive, and may obsucre the actual functional distance rather than delivering more functional information. Therefore, we only used continuous morphological traits and quantified the Euclidean distance for pairwise fish species to represent their functional distance in this study.

### 2.4 Calculation of phylogenetic and functional measures

Firstly, based upon the pairwise phylogenetic and functional distances, we calculated two phylogenetic and functional diversity metrics used widely for the native fishes in each lake using the *picante* package (Kembel et al., 2010): the mean phylogenetic and functional distances (MPD & MFD), as well as the mean nearest phylogenetic and functional distances (MNTD & MNFD) (Pinto‐Ledezma et al., 2020; Webb et al., 2002). Then, for each species introduced into each lake, we calculated two metrics to measure the phylogenetic and functional similarities between the introduced and native species (Li, Cadotte, et al., 2015; Strauss et al., 2006): (1) the introduced‐native mean phylogenetic and functional distances (I‐N MPD & I‐N MFD), which measures the mean distance between each introduced fish species and all native fishes, and (2) the introduced‐native nearest phylogenetic and functional distances (I‐N MNTD & I‐N MNFD), which measures the distance between each introduced fish species and the most evolutionarily related or most functionally similar native species.

### 2.5 Phylogenetic signal and correlation between phylogenetic and functional measures

We tested the phylogenetic signal for the functional traits using the *Mantel test* with the *ape* package (Emmanuel et al., 2004), to check whether more closely related species have more similar functional traits. We also examined the correlations between native MPD and MFD, as well as I‐N MPD and I‐N MFD. We found a significant phylogenetic signal (Figure 2a) and a significant correlation between MPD and MFD (Figure 2b), and I‐N MPD and I‐N MFD (Figure 2c). However, the relatively low correlation coefficients implied that the phylogenetic pattern captures limited functional information. We verified further that including both phylogenetic and functional metrics did not lead to multicollinearity issues given that the variance inflation factors (VIFs) for all of them were less than three. Therefore, we integrated both functional and phylogenetic metrics into one model in the following analyses to distinguish their effects on the establishment of exotic fishes.

### 2.6 Statistical analyses

We used three complementary statistical methods: (1) the Bayesian hierarchical model includes the phylogenetic covariance matrix in the model to account conveniently for the phylogenetic non‐independence among introduced species; (2) model averaging of generalized linear mixed models (GLMMs) allows us to compare different models conveniently and obtain the best model fit to the data; and (3) structural equation modeling (SEM) can help identify the direct and indirect effects of multiple predictive variables on establishment success.

#### 2.6.1 Bayesian phylogenetic hierarchical models

We included the I‐N MPD, I‐N MFD, native MPD, native MFD, native species richness, lake area, elevation, and latitude as fixed effects to predict successful or failed introductions with a Bernoulli error structure. The lake name and introduced species were used as random effects to account for the statistical non‐independence of multiple introductions within a specific lake, and of multiple introductions of a given species. We considered phylogenetic non‐independence among introduced species by incorporating the phylogenetic covariance structure into the model. We derived the phylogenetic covariance matrix of the entire phylogeny using the *ape* package (Emmanuel et al., 2004) and extracted the sub‐matrix corresponding to the 23 introduced species. The model was fitted using the *INLA* package (Rue et al., 2009), which uses integrated nested Laplace approximation for Bayesian inference. This method approximates Bayesian posterior distributions quickly and accurately without using MCMC, and allows for complex layered random effects, including autocorrelation terms (Rue et al., 2017). We used the default INLA priors for all of the fixed effect parameters and the log‐gamma prior (with *shape* = 0.01 and *inverse scale* = 0.01) for the hyperparameters (random effect parameters). All of the continuous predictor variables were standardized before they were entered into the model using the z‐score (by subtracting the mean and dividing by the standard deviation) to interpret parameter estimates on a comparable scale. We evaluated the relative importance of predictors in influencing establishment success by calculating the ratio between the absolute value of the parameter estimates for each of the predictors and the sum of all parameter estimates in the model. This method is similar to a variance partitioning analysis because the standardized variables have been used in the model and have been adopted in several studies (Gross et al., 2017; Le Provost et al., 2020).

#### 2.6.2 Model averaging in the generalized linear mixed models

Firstly, we fitted a full generalized linear mixed model (GLMM) with the *lme4* package using the same fixed and random effects as in the Bayesian model (Bates et al., 2015). Then, we performed a multi‐model comparison using the *dredge* function of *MuMIn* package (Barton, 2022), which constructs all possible candidate sub‐models and ranks them according to the corrected Akaike Information Criterion (AICc) values. Finally, we performed a model averaging for the best models with ΔAICc <2 using the *model.avg* function in the *MuMIn* package, and derived the best predictor variables as well as associated coefficients. Based upon the averaging model, we evaluated the effects of predictive variables and compared them to the Bayesian model.

#### 2.6.3 Structural equation modeling

To evaluate the direct and indirect effects of diversity and similarity on establishment success and assess the influence of geographical factors (elevation, latitude, and area), we adopted piecewise structural equation modeling (SEM) (Shipley, 2009) using the *piecewiseSEM* package (Lefcheck, 2016). Piecewise SEM can integrate a set of component models including random effects, and allow for non‐Gaussian error distributions, and is thus appropriate for our data structure. Specifically, we incorporated the lake and introduced species as random effects in each component model to account for sampling non‐independence. Multicollinearity in each component model was checked according to the VIF. Model fits were evaluated using the Fisher's C statistic based upon Shipley's *d‐sep* test (Shipley, 2000), and model selection was performed using the AIC statistic for the *d‐sep* test (Shipley, 2013). Standardized path coefficients for the linear mixed component models and the *R*2 for the endogenous variables were calculated directly using the *piecewiseSEM* package. For the generalized linear mixed component model, we extended the method used by *piecewiseSEM* for the generalized linear model and calculated the standardized path coefficients manually by including the standard variance of random effects. Based upon our prior knowledge, we constructed an initial SEM (Figure S1), and based upon the *d‐sep* test, we added missing paths step‐by‐step to construct a set of candidate SEMs. We selected the best model with the lowest AIC value and calculated the direct, indirect, and relative total effects on establishment success. We also developed another SEM to evaluate the effect of water temperature specifically on establishment success and its influence on diversity and similarity by replacing the geographical factors with the accumulated and maximum water temperature, respectively.

We performed the analyses using both mean distance indices (MPD, MFD, I‐N MPD, and I‐N MFD) and nearest distance indices (MNTD, MNFD, I‐N MNTD, and I‐N MNFD). The results were compared by the Watanabe Akaike information criterion (WAIC) (Watanabe & Opper, 2010), which follows a more fully Bayesian approach to construct an information criterion. Because the model that used mean phylogenetic and functional metrics (WAIC = 1158.1) was superior to the nearest metrics (WAIC = 1168.8), we report only the results of the mean distance indices in the main text, and the results on the nearest metrics are presented in the supplementary information.

## 3 RESULTS

**FIGURE 3:**
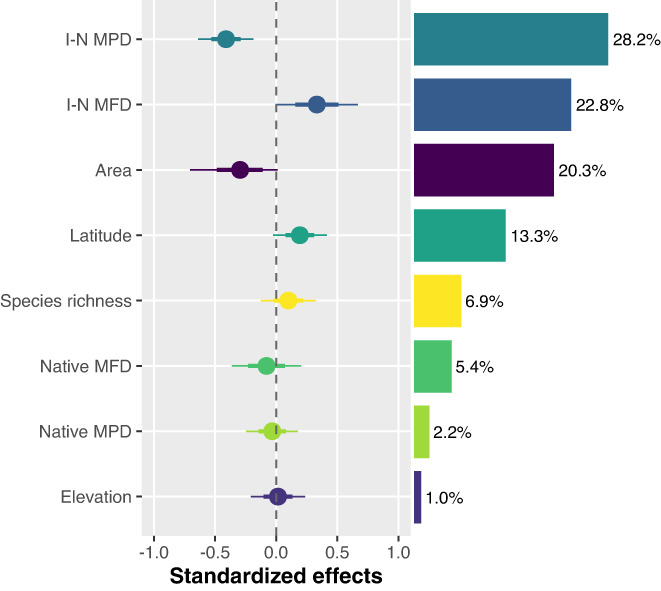
The effects and relative importance of predictive variables in explaining the establishment success of exotic fishes. The standardized effect size (±95% and 70% credible intervals) was derived from the Bayesian hierarchical model with predictive variables standardized by subtracting the mean and dividing by the standard deviation. The relative importance of each predictor, expressed as the percentage of explained variance, was calculated using the ratio between its standardized parameter estimate and the sum of all standardized parameter estimates. Dashed lines indicate effect = 0. Positive effects (the 95% credible interval does not include zero) indicate that the probability of establishment increases with increasing values of predictive variables, while the negative effects indicate the converse.

**FIGURE 4:**
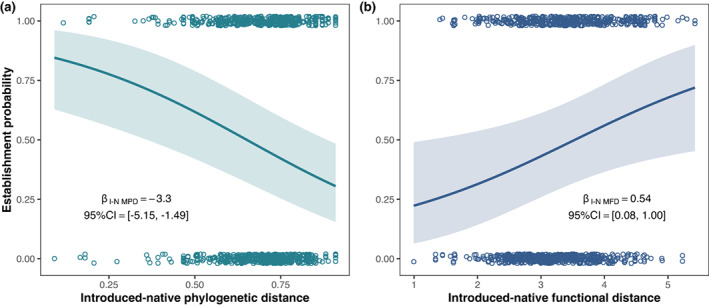
The respective relationships between the probability of successful establishment and introduced‐native mean phylogenetic distance (a), and introduced‐native mean functional distance (b). The predictive curves (with 95% credible bands) were derived from the Bayesian hierarchical models with the other predictive variables fixed at the mean values. To make the relationships straightforward, the models were fitted with the raw, rather than standardized, values of predictive variables.

The Bayesian phylogenetic hierarchical models showed that the phylogenetic and functional similarities accounted for 51.0% of explained variance, the geographical characteristics (lake area, latitude, and elevation) explained 34.5%, and the taxonomic, phylogenetic, and functional diversity explained the remaining 14.5% (Figure 3). Specifically, the introduced‐native phylogenetic distances had a significant negative effect on successful establishment, and accounted for 28.2% of explained variance (Figures 3, 4; Table S1), while the introduced‐native functional distances had a significant positive effect, and accounted for 22.8% of explained variance (Figures 3, 4; Table S1). The opposing significant effects of phylogenetic and functional similarities were confirmed by the model average for the generalized linear mixed models (GLMMs) (Table S2).

**FIGURE 5:**
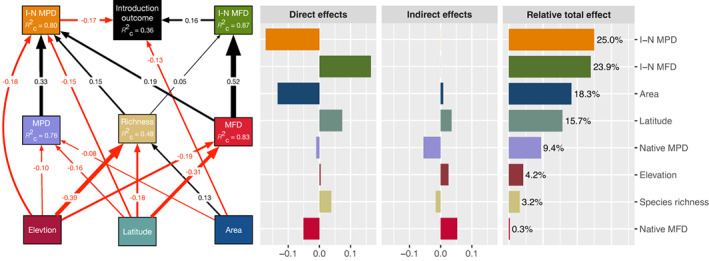
Structural equation modeling (SEM) performed to explore the direct and indirect effects of similarity, diversity, and geographical factors on the establishment of exotic fish species. MPD denotes the mean phylogenetic distance of native species, while MFD denotes the mean functional distance (i.e., phylogenetic and functional diversity, respectively). I‐N MPD denotes the mean phylogenetic distance between introduced exotic species and native species, while the I‐N MFD denotes the mean functional distance between introduced exotic species and native species (i.e., exotic–native phylogenetic and functional similarities, respectively). This SEM is determined through model comparison and fits the data well (Fisher's *C* = 0.61, d.f. = 4, *p* = .96; *K* = 49, *n* = 965). For clarity, only the significant paths (*p* < .05) are shown in the figure. Boxes represent measured variables and arrows represent relationships among variables. Black arrows denote positive relationships and red arrows negative associations. Standardized path coefficients are given for each significant path, the width of which is scaled by the magnitude of the standardized path coefficient. The conditional *R*c2 (based upon both fixed and random effects) for each endogenous variable is reported in the corresponding boxes. The direct, indirect, and relative total effects on the establishment of exotic species are calculated and shown in the right part of this figure.

Based upon the best structural equation modeling (Tables S3, S4), the introduced‐native phylogenetic and functional distances had direct negative and positive effects on establishment success, and accounted for 25.0% and 23.9% of the total effect, respectively (Figure 5). Lake area had a direct negative effect on establishment and meanwhile influenced the establishment indirectly by affecting native species and phylogenetic diversity, and accounted for 18.3% of the total effect (Figure 5). Latitude and elevation had indirect effects on the establishment by influencing native diversity, and accounted for 15.7% and 4.2% of the total effect, respectively. Taxonomic, phylogenetic, and functional diversity influenced establishment indirectly by adjusting the introduced‐native phylogenetic and functional distances, and accounted for 3.2%, 9.4%, and 0.3% of the total effect, respectively (Figure 5). Even considering the influence of water temperature, mean phylogenetic and functional distance still had significant and opposing direct effects on establishment success (Figure S2).

## 4 DISCUSSION

Using a unique dataset on the successful and failed establishment of fishes introduced into freshwater lakes, we tested the effects of exotic–native phylogenetic and functional similarities on their establishment success. Our main finding is that phylogenetic relatedness promoted successful establishment of exotic fishes, while functional similarity hampered success. The opposing effects of phylogenetic and functional similarities indicate that a successful invader should be phylogenetically closely related, but functionally distant to natives. This finding suggests that both aspects of Darwin's naturalization conundrum may operate simultaneously, but are characterized by phylogenetic and functional dimensions, respectively.

The possible explanation of this contrasting effect is that phylogenetic and functional similarities offer complementary, rather than similar information, in which the former largely reflects the pre‐adaptation to novel environments and the latter characterizes the competition between exotic and resident species. Functional similarity has been used commonly as a surrogate of phylogenetic relatedness to evaluate Darwin's naturalization conundrum (Divíšek et al., 2018; Pinto‐Ledezma et al., 2020; Rocha & Cianciaruso, 2021). However, several studies have shown that phylogenetic and functional distances were not congruent (Cadotte et al., 2019; Mazel et al., 2018) and may provide different information about species differences and associated patterns of coexistence (Cadotte et al., 2013, 2017). We also found that, although there was a significant phylogenetic signal for the phenotypic traits, exotic–native phylogenetic distance explained only approximately 25% of the variation in functional distance (Figure 2). This indicates that the phylogenetic niche with respect to these phenotypic traits may be less conserved (Losos, 2008) and similarity in the phylogenetic and functional dimensions may capture different information in predicting the success of exotic species (Zheng et al., 2018). Specifically, phylogenetic similarity, as an integrative measure of all the traits, may capture the similarity in some important functions that we have yet to discover with commonly used phenotypical traits. For example, phylogenetic similarity may capture some similarities in physiological traits such as thermal tolerance (Comte & Olden, 2017) and hypoxia tolerance (Collins et al., 2013), which may influence the establishment of exotic fishes in novel environments. Consequently, on the one hand, phylogenetically closely related exotics may adapt better to environmental factors because of physiological tolerance more similar to that of natives, consistent with PAH. On the other hand, similar phenotypic traits such as body size and oral gape position may mean similar food or trophic level to natives, and thus suffer stronger resource competition with natives, in accordance with DNH.

Overall, we suggest that the opposing patterns arise from asymmetry in phylogenetic and functional similarities. Phylogenetic similarity, which represents some important functions that are not captured by measured phenotypical traits, allows exotic species to pre‐adapt better to the novel abiotic environment or escape from predators. However, to become established successfully, they need to possess some distinct traits to reduce their competition with native species. Although we did not test these potential processes explicitly in this study, our results indicate that using either aspect of ecological similarity alone to explore Darwin's conundrum may yield biased conclusions and that integrating phylogenetic and functional dimensions is required to better resolve the conundrum. We advocate future experimental studies that select exotic species that are phylogenetically close relatives to natives, but possess distinct traits, and the converse, to further examine the mechanisms underlying the opposing effects of phylogenetic and functional similarities on invasion success.

Another important finding of our study is that, compared with native diversity and abiotic factors, exotic–native ecological similarities play more important roles in the establishment of exotic fishes, and account for approximately half of the explained variance. While most studies that have tested Darwin's naturalization conundrum have failed to reveal the relative importance of similarity, one study in the plant community obtained results similar to ours. Carboni et al. (2016) showed that, compared with functional characteristics and introduction history, phylogenetic and functional similarities were the most important determinants of successful invasion (Carboni et al., 2016). Our results indicate that ecological similarities may have far more important effects on invasion success than we expected and highlight the importance of considering these similarities to predict biological invasions. Native biodiversity has long been proposed to be a barrier to invasion success, given that exotic species are more likely to suffer strong competition in diverse communities (Elton, 1958). However, we showed that diversity (regardless of the taxonomic, phylogenetic, and functional dimensions) had no significant direct effects on exotic fish establishment when the effect of exotic–native similarities was considered. Rather, they simply played an indirect role by adjusting the strength of similarities. Consistent with our results, one experimental study of bacteria communities also found that similarity rather than diversity predicted invasive ability better (Tan et al., 2015). We also found that, because high diversity decreases phylogenetic and functional similarities simultaneously (i.e., increases phylogenetic and functional distances, Figure 5), the total effect of diversity on establishment depended upon the relative extent of its influence on both aspects. If increasing diversity decreases phylogenetic similarity more than functional similarity, high diversity would be more likely to hamper establishment. Otherwise, it would promote success. This provides an alternative explanation for the invasion paradox (i.e., the opposing diversity‐invasibility relationships) (Fridley et al., 2007).

We found that lake area had a direct negative effect on successful establishment in addition to an indirect mediation effect by influencing native diversity, which is consistent with a study conducted in freshwater lakes (Henriksson, Wardle, et al., 2016). We suggest that a large lake area may denote a more heterogeneous environment and more predators for the introduced fishes, which potentially lowers the probability of successful establishment. As expected, we also showed that native diversity decreased significantly at high elevation and latitude, while increasing with rising water temperature. While decreasing diversity increases phylogenetic similarity and thus promotes the establishment of exotics fishes at high elevations and latitudes, it also increases the functional similarity and hampers establishment simultaneously. Taken together, these abiotic factors primarily play indirect roles in exotic fish establishment by mediating diversity and similarities, and their net effects on establishment success depend upon how strongly they influence both aspects of similarity.

Several limitations of our study should be acknowledged. First, the choice of traits may influence the functional distances calculated and different traits may affect establishment success differentially (Kraft et al., 2015). While the ten morphological traits that were widely used were combined in our study to calculate functional distance, it is unknown to what extent the results may differ when other traits, such as physiological and reproductive characteristics, are considered. Merging these traits with morphological traits in the future is needed to provide a better understanding on the functional structure of fish community and its influence on fish invasions (Brosse et al., 2021). In addition, it should be noted that these morphological traits were measured from limited fish individuals per species and intraspecific variations may have some influence on the generalization of our results. Second, given that all fish introductions in this study were intentional and mainly for purposes of consumption and recreation, some species with specific traits may be preferred for introduction, with more numbers of individuals and more frequent introduction events (i.e., high propagule pressure). This could result in more opportunities for them to become established, potentially obscuring our results based on competition and environmental adaptation (Ricciardi & Mottiar, 2006). Finally, while biological invasions can be divided into different stages, i.e., introduction, establishment/naturalization, spread, and impact (Richardson et al., 2000), the effects of phylogenetic and functional similarities often depend upon these specific stages (Divíšek et al., 2018; Li, Guo, et al., 2015). Our study focuses only on the establishment stage, and more studies along the introduction–naturalization–invasion continuum are still required to obtain a full understanding of biological invasions.

In summary, our work provides a critical empirical assessment of the roles of ecological similarity, diversity, geographical, and climate factors in the establishment of exotic fishes. We demonstrated that phylogenetic and functional similarities were the most important predictors of establishment relative to other biotic and abiotic factors, and highlighted the importance of considering ecological similarities to predict biological invasions. The opposing effects of phylogenetic relatedness and functional similarity provide a novel insight into reconciling Darwin's naturalization conundrum. In the future, a key step forward is to identify which fish traits represent environmental adaptation, predator susceptibility, and whether those traits are conserved phylogenetically or not, which will offer a mechanistic understanding of the contrasting effects of phylogenetic and functional dimensions.

## AUTHOR CONTRIBUTIONS

MX conceived the study design, conducted the analyses, and wrote the first draft of the manuscript. SL and JD interpreted the results and edited the following versions. XM, DG, MF, YY and YH contributed substantially to revisions.

## CONFLICT OF INTEREST

The authors declare that they have no competing interests.

## Supporting information


Data S1

Data S2

## Data Availability

The data that support the findings of this study are available in the Dryad Digital Repository (https://doi.org/10.5061/dryad.vx0k6djvh).

## References

[gcb16360-bib-0001] Barton, K. (2022). MuMIn: Multi‐model inference. R package version 1.46.0. https://cran.r-project.org/package=MuMIn

[gcb16360-bib-0002] Bates, D. , Maechler, M. , Bolker, B. , & Walker, S. (2015). Fitting liner mixed‐effects models using lme4. Journal of Statistical Software, 67(1), 1–48. 10.18637/jss.v067.i01

[gcb16360-bib-0003] Beaury, E. M. , Finn, J. T. , Corbin, J. D. , Barr, V. , & Bradley, B. A. (2020). Biotic resistance to invasion is ubiquitous across ecosystems of the United States. Ecology Letters, 23(3), 476–482. 10.1111/ele.13446 31875651

[gcb16360-bib-0004] Bezeng, S. B. , Davies, J. T. , Yessoufou, K. , Maurin, O. , Van Der Bank, M , & Fridley, J. (2015). Revisiting Darwin's naturalization conundrum: Explaining invasion success of non‐native trees and shrubs in southern Africa. Journal of Ecology, 103(4), 871–879. 10.1111/1365-2745.12410

[gcb16360-bib-0005] Brosse, S. , Charpin, N. , Su, G. , Toussaint, A. , Herrera‐R, G. A. , Tedesco, P. A. , Villéger, S. , & Blowes, S. (2021). FISHMORPH: A global database on morphological traits of freshwater fishes. Global Ecology and Biogeography, 30(12), 2330–2336. 10.1111/geb.13395

[gcb16360-bib-0006] Cadotte, M. , Albert, C. H. , & Walker, S. C. (2013). The ecology of differences: Assessing community assembly with trait and evolutionary distances. Ecology Letters, 16(10), 1234–1244. 10.1111/ele.12161 23910526

[gcb16360-bib-0007] Cadotte, M. W. , Campbell, S. E. , Li, S. P. , Sodhi, D. S. , & Mandrak, N. E. (2018). Preadaptation and naturalization of nonnative species: Darwin's two fundamental insights into species invasion. Annual Review of Plant Biology, 69(1), 661–684. 10.1146/annurev-arplant-042817-040339 29489400

[gcb16360-bib-0008] Cadotte, M. W. , Carboni, M. , Si, X. , Tatsumi, S. , & Gibson, D. (2019). Do traits and phylogeny support congruent community diversity patterns and assembly inferences? Journal of Ecology, 107(5), 2065–2077. 10.1111/1365-2745.13247

[gcb16360-bib-0009] Cadotte, M. W. , Davies, T. J. , & Peres‐Neto, P. R. (2017). Why phylogenies do not always predict ecological differences. Ecological Monographs, 87(4), 535–551. 10.1002/ecm.1267

[gcb16360-bib-0010] Carboni, M. , Munkemuller, T. , Lavergne, S. , Choler, P. , Borgy, B. , Violle, C. , Essl, F. , Roquet, C. , Munoz, F. , Divgrass, C. , & Thuiller, W. (2016). What it takes to invade grassland ecosystems: Traits, introduction history and filtering processes. Ecology Letters, 19(3), 219–229. 10.1111/ele.12556 26689431 PMC4972145

[gcb16360-bib-0011] Catford, J. A. , Jansson, R. , & Nilsson, C. (2009). Reducing redundancy in invasion ecology by integrating hypotheses into a single theoretical framework. Diversity and Distributions, 15(1), 22–40. 10.1111/j.1472-4642.2008.00521.x

[gcb16360-bib-0012] Cavender‐Bares, J. , Kozak, K. H. , Fine, P. V. , & Kembel, S. W. (2009). The merging of community ecology and phylogenetic biology. Ecology Letters, 12(7), 693–715. 10.1111/j.1461-0248.2009.01314.x 19473217

[gcb16360-bib-0013] Collins, G. M. , Clark, T. D. , Rummer, J. L. , & Carton, A. G. (2013). Hypoxia tolerance is conserved across genetically distinct sub‐populations of an iconic, tropical Australian teleost (*Lates calcarifer*). Conservation physiology, 1(1), cot029. 10.1093/conphys/cot029 27293613 PMC4806625

[gcb16360-bib-0014] Comte, L. , & Olden, J. D. (2017). Evolutionary and environmental determinants of freshwater fish thermal tolerance and plasticity. Global Change Biology, 23(2), 728–736. 10.1111/gcb.13427 27406402

[gcb16360-bib-0015] Cuthbert, R. N. , Pattison, Z. , Taylor, N. G. , Verbrugge, L. , Diagne, C. , Ahmed, D. A. , Leroy, B. , Angulo, E. , Briski, E. , & Capinha, C. (2021). Global economic costs of aquatic invasive alien species. Science of the Total Environment, 775, 145238. 10.1016/j.scitotenv.2021.145238 33715860

[gcb16360-bib-0016] Daehler, C. C. (2001). Darwin's naturalization hypothesis revisited. The American Naturalist, 158(3), 324–330. 10.1086/321316 18707328

[gcb16360-bib-0017] Dawson, W. , Moser, D. , Van Kleunen, M. , Kreft, H. , Pergl, J. , Pyšek, P. , Weigelt, P. , Winter, M. , Lenzner, B. , & Blackburn, T. M. (2017). Global hotspots and correlates of alien species richness across taxonomic groups. Nature Ecology and Evolution, 1, 0186. 10.1038/s41559-017-0186

[gcb16360-bib-0018] De Bello, F. , Šmilauer, P. , Diniz‐Filho, J. a. F. , Carmona, C. P. , Lososová, Z. , Herben, T. , & Götzenberger, L. (2017). Decoupling phylogenetic and functional diversity to reveal hidden signals in community assembly. Methods in Ecology and Evolution, 8(10), 1200–1211. 10.1111/2041-210X.12735

[gcb16360-bib-0019] Diez, J. M. , Sullivan, J. J. , Hulme, P. E. , Edwards, G. , & Duncan, R. P. (2008). Darwin's naturalization conundrum: Dissecting taxonomic patterns of species invasions. Ecology Letters, 11(7), 674–681. 10.1111/j.1461-0248.2008.01178.x 18400019

[gcb16360-bib-0020] Divíšek, J. , Chytrý, M. , Beckage, B. , Gotelli, N. J. , Lososová, Z. , Pyšek, P. , Richardson, D. M. , & Molofsky, J. (2018). Similarity of introduced plant species to native ones facilitates naturalization, but differences enhance invasion success. Nature Communications, 9(1), 4631. 10.1038/s41467-018-06995-4 PMC6219509 30401825

[gcb16360-bib-0021] Duncan, R. P. , & Williams, P. A. (2002). Darwin's naturalization hypothesis challenged. Nature, 417(6889), 608–609. 10.1038/417608a 12050652

[gcb16360-bib-0022] Edgar, R. C. (2004). MUSCLE: Multiple sequence alignment with high accuracy and high throughput. Nucleic Acids Research, 32(5), 1792–1797. 10.1093/nar/gkh340 15034147 PMC390337

[gcb16360-bib-0023] Elton, C. S. (1958). The ecology of invasions by animals and plants. University of Chicago Press.

[gcb16360-bib-0024] Emmanuel, P. , Julien, C. , & Korbinian, S. (2004). APE: Analyses of phylogenetics and evolution in R language. Bioinformatics, 20(2), 289–290. 10.1093/bioinformatics/btg412 14734327

[gcb16360-bib-0025] Enders, M. , Havemann, F. , Ruland, F. , Bernard‐Verdier, M. , Catford, J. A. , Gómez‐Aparicio, L. , Haider, S. , Heger, T. , Kueffer, C. , Kühn, I. , Meyerson, L. A. , Musseau, C. , Novoa, A. , Ricciardi, A. , Sagouis, A. , Schittko, C. , Strayer, D. L. , Vilà, M. , Essl, F. , … Belmaker, J. (2020). A conceptual map of invasion biology: Integrating hypotheses into a consensus network. Global Ecology and Biogeography, 29(6), 978–991. 10.1111/geb.13082 34938151 PMC8647925

[gcb16360-bib-0026] Feng, Y. , Fouqueray, T. D. , & Van Kleunen, M. (2019). Linking Darwin's naturalisation hypothesis and Elton's diversity–Invasibility hypothesis in experimental grassland communities. Journal of Ecology, 107(2), 794–805. 10.1111/1365-2745.13061

[gcb16360-bib-0027] Fridley, J. , Stachowicz, J. , Naeem, S. , Sax, D. , Seabloom, E. , Smith, M. , Stohlgren, T. , Tilman, D. , & Holle, B. V. (2007). The invasion paradox: Reconciling pattern and process in species invasions. Ecology, 88(1), 3–17. 10.1890/0012-9658(2007)88[3:tiprpa]2.0.co;2 17489447

[gcb16360-bib-0028] Fristoe, T. S. , Chytrý, M. , Dawson, W. , Essl, F. , Heleno, R. , Kreft, H. , Maurel, N. , Pergl, J. , Pyšek, P. , & Seebens, H. (2021). Dimensions of invasiveness: Links between local abundance, geographic range size, and habitat breadth in Europe's alien and native floras. Proceedings of the National Academy of Sciences of the United States of America, 118(22), e2021173118. 10.1073/pnas.2021173118 34050023 PMC8179145

[gcb16360-bib-0029] Gallien, L. , & Carboni, M. (2017). The community ecology of invasive species: Where are we and what's next? Ecography, 40(2), 335–352. 10.1111/ecog.02446

[gcb16360-bib-0030] Gower, J. C. (1971). A general coefficient of similarity and some of its properties. Biometrics, 27(4), 857–871. 10.2307/2528823

[gcb16360-bib-0031] Gross, N. , Le Bagousse‐Pinguet, Y. , Liancourt, P. , Berdugo, M. , Gotelli, N. J. , & Maestre, F. T. (2017). Functional trait diversity maximizes ecosystem multifunctionality. Nature Ecology and Evolution, 1, 0132. 10.1038/s41559-017-0132 28497123 PMC5421574

[gcb16360-bib-0032] Henriksson, A. , Rydberg, C. , & Englund, G. (2016). Failed and successful intentional introductions of fish species into 821 Swedish lakes. Ecology, 97(5), 1364. 10.1890/15-1707.1

[gcb16360-bib-0033] Henriksson, A. , Wardle, A. , Trygg, J. , Diehl, S. , & Englund, G. (2016). Strong invaders are strong defenders‐implications for the resistance of invaded communities. Ecology Letters, 19(4), 487–494. 10.1111/ele.12586 26947421

[gcb16360-bib-0034] Jiang, L. , Tan, J. , & Pu, Z. (2010). An experimental test of Darwin's naturalization hypothesis. The American Naturalist, 175(4), 415–423. 10.1086/650720 20170339

[gcb16360-bib-0035] Kembel, S. W. , Cowan, P. D. , Helmus, M. R. , Cornwell, W. K. , Morlon, H. , Ackerly, D. D. , Blomberg, S. P. , & Webb, C. O. (2010). Picante: R tools for integrating phylogenies and ecology. Bioinformatics, 26(11), 1463–1464. 10.1093/bioinformatics/btq166 20395285

[gcb16360-bib-0036] Kolar, C. S. , & Lodge, D. M. (2001). Progress in invasion biology: Predicting invaders. Trends in Ecology and Evolution, 16(4), 199–204. 10.1016/s0169-5347(01)02101-2 11245943

[gcb16360-bib-0037] Kraft, N. J. , Godoy, O. , & Levine, J. M. (2015). Plant functional traits and the multidimensional nature of species coexistence. Proceedings of the National Academy of Sciences of the United States of America, 112(3), 797–802. 10.1073/pnas.1413650112 25561561 PMC4311865

[gcb16360-bib-0038] Le Provost, G. , Badenhausser, I. , Le Bagousse‐Pinguet, Y. , Clough, Y. , Henckel, L. , Violle, C. , Bretagnolle, V. , Roncoroni, M. , Manning, P. , & Gross, N. (2020). Land‐use history impacts functional diversity across multiple trophic groups. Proceedings of the National Academy of Sciences of the United States of America, 117(3), 1573–1579. 10.1073/pnas.1910023117 31907310 PMC6983382

[gcb16360-bib-0039] Lefcheck, J. S. (2016). piecewiseSEM: Piecewise structural equation modelling in r for ecology, evolution, and systematics. Methods in Ecology and Evolution, 7(5), 573–579. 10.1111/2041-210X.12512

[gcb16360-bib-0040] Li, S. , Cadotte, M. W. , Meiners, S. J. , Hua, Z. , Shu, H. , Li, J. , & Shu, W. (2015). The effects of phylogenetic relatedness on invasion success and impact: Deconstructing Darwin's naturalisation conundrum. Ecology Letters, 18(12), 1285–1292. 10.1111/ele.12522 26437879

[gcb16360-bib-0041] Li, S. , Guo, T. , Cadotte, M. W. , Chen, Y.‐J. , Kuang, J.‐L. , Hua, Z.‐S. , Zeng, Y. , Song, Y. , Liu, Z. , Shu, W.‐S. , Li, J.‐T. , & Barlow, J. (2015). Contrasting effects of phylogenetic relatedness on plant invader success in experimental grassland communities. Journal of Applied Ecology, 52(1), 89–99. 10.1111/1365-2664.12365

[gcb16360-bib-0042] Losos, J. B. (2008). Phylogenetic niche conservatism, phylogenetic signal and the relationship between phylogenetic relatedness and ecological similarity among species. Ecology Letters, 11(10), 995–1003. 10.1111/j.1461-0248.2008.01229.x 18673385

[gcb16360-bib-0043] Marx, H. E. , Giblin, D. E. , Dunwiddie, P. W. , & Tank, D. C. (2016). Deconstructing Darwin's naturalization conundrum in the San Juan Islands using community phylogenetics and functional traits. Diversity and Distributions, 22(3), 318–331. 10.1111/ddi.12401

[gcb16360-bib-0044] Mayfield, M. M. , & Levine, J. M. (2010). Opposing effects of competitive exclusion on the phylogenetic structure of communities. Ecology Letters, 13(9), 1085–1093. 10.1111/j.1461-0248.2010.01509.x 20576030

[gcb16360-bib-0045] Mazel, F. , Pennell, M. W. , Cadotte, M. W. , Diaz, S. , Dalla Riva, G. V. , Grenyer, R. , Leprieur, F. , Mooers, A. O. , Mouillot, D. , & Tucker, C. M. (2018). Prioritizing phylogenetic diversity captures functional diversity unreliably. Nature Communications, 9(1), 2888. 10.1038/s41467-018-05126-3 PMC6056549 30038259

[gcb16360-bib-0046] Ordonez, A. (2014). Functional and phylogenetic similarity of alien plants to co‐occurring natives. Ecology, 95(5), 1191–1202. 10.1890/13-1002.1 25000751

[gcb16360-bib-0047] Park, D. S. , Feng, X. , Maitner, B. S. , Ernst, K. C. , & Enquist, B. J. (2020). Darwin's naturalization conundrum can be explained by spatial scale. Proceedings of the National Academy of Sciences of the United States of America, 117(20), 10904–10910. 10.1073/pnas.1918100117 32366659 PMC7245080

[gcb16360-bib-0048] Pearson, D. E. , Ortega, Y. K. , Eren, O. , & Hierro, J. L. (2018). Community assembly theory as a framework for biological invasions. Trends in Ecology and Evolution, 33(5), 313–325. 10.1016/j.tree.2018.03.002 29605085

[gcb16360-bib-0049] Pinto‐Ledezma, J. N. , Villalobos, F. , Reich, P. B. , Catford, J. A. , Larkin, D. J. , & Cavender‐Bares, J. (2020). Testing Darwin's naturalization conundrum based on taxonomic, phylogenetic, and functional dimensions of vascular plants. Ecological Monographs, 90(4), e01420. 10.1002/ecm.1420

[gcb16360-bib-0050] Posada, D. (2008). jModelTest: Phylogenetic model averaging. Molecular Biology and Evolution, 25(7), 1253–1256. 10.1093/molbev/msn083 18397919

[gcb16360-bib-0051] Procheş, Ş. , Wilson, J. R. , Richardson, D. M. , & Rejmánek, M. (2008). Searching for phylogenetic pattern in biological invasions. Global Ecology and Biogeography, 17(1), 5–10. 10.1111/j.1466-8238.2007.00333.x

[gcb16360-bib-0052] Pyšek, P. , Hulme, P. E. , Simberloff, D. , Bacher, S. , Blackburn, T. M. , Carlton, J. T. , Dawson, W. , Essl, F. , Foxcroft, L. C. , & Genovesi, P. (2020). Scientists' warning on invasive alien species. Biological Reviews, 95(6), 1511–1534. 10.1111/brv.12627 32588508 PMC7687187

[gcb16360-bib-0053] Pyšek, P. , & Richardson, D. M. (2010). Invasive species, environmental change and management, and health. Annual Review of Environment and Resources, 35, 25–55. 10.1146/annurev-environ-033009-095548

[gcb16360-bib-0054] Qian, H. , Sandel, B. , & Meireles, J. E. (2021). Darwin's preadaptation hypothesis and the phylogenetic structure of native and alien regional plant assemblages across North America. Global Ecology and Biogeography, 31(3), 531–545. 10.1111/geb.13445

[gcb16360-bib-0055] Remco, B. , Heled, J. , Kühnert, D. , Vaughan, T. , Wu, C. , Xie, D. , Suchard, M. , Rambaut, A. , & Drummond, A. (2014). BEAST 2: A software platform for Bayesian evolutionary analysis. PLoS Computational Biology, 10(4), e1003537. 10.1371/journal.pcbi.1003537 24722319 PMC3985171

[gcb16360-bib-0056] Ricciardi, A. , & Mottiar, M. (2006). Does Darwin's naturalization hypothesis explain fish invasions? Biological Invasions, 8(6), 1403–1407. 10.1007/s10530-006-0005-6

[gcb16360-bib-0057] Richardson, D. M. , Pyšek, P. , Rejmánek, M. , Barbour, M. G. , Panetta, F. D. , & West, C. J. (2000). Naturalization and invasion of alien plants: Concepts and definitions. Diversity and Distributions, 6(2), 93–107. 10.1046/j.1472-4642.2000.00083.x

[gcb16360-bib-0058] Rocha, B. S. , & Cianciaruso, M. V. (2021). Water temperature and lake size explain Darwin's conundrum for fish establishment in boreal lakes. Hydrobiologia, 848(9), 2033–2042. 10.1007/s10750-020-04434-4

[gcb16360-bib-0059] Rue, H. , Riebler, A. , Sorbye, S. H. , Illian, J. B. , Simpson, D. P. , & Lindgren, F. K. (2017). Bayesian computing with INLA: A review. Annual Review of Statistics and Its Application, 4(1), 395–421. 10.1146/annurev-statistics-060116-054045

[gcb16360-bib-0060] Rue, H. , Sara, M. , & Nicolas, C. (2009). Approximate Bayesian inference for latent Gaussian models by using integrated nested Laplace approximations. Journal of the Royal Statistical Society, 71(2), 319–392. 10.1111/j.1467-9868.2008.00700.x

[gcb16360-bib-0061] Seebens, H. , Bacher, S. , Blackburn, T. M. , Capinha, C. , Dawson, W. , Dullinger, S. , Genovesi, P. , Hulme, P. E. , Van Kleunen, M. , & Kühn, I. (2021). Projecting the continental accumulation of alien species through to 2050. Global Change Biology, 27(5), 970–982. 10.1111/gcb.15333 33000893

[gcb16360-bib-0062] Seebens, H. , Blackburn, T. M. , Dyer, E. E. , Genovesi, P. , Hulme, P. E. , Jeschke, J. M. , Pagad, S. , Pyšek, P. , Van Kleunen, M. , & Winter, M. (2018). Global rise in emerging alien species results from increased accessibility of new source pools. Proceedings of the National Academy of Sciences of the United States of America, 115(10), E2264–E2273. 10.1073/pnas.1719429115 29432147 PMC5877962

[gcb16360-bib-0063] Shipley, B. (2000). A new inferential test for path models based on directed acyclic graphs. Structural Equation Modeling, 7(2), 206–218. 10.1207/S15328007SEM0702_4

[gcb16360-bib-0064] Shipley, B. (2009). Confirmatory path analysis in a generalized multilevel context. Ecology, 90(2), 363–368. 10.1890/08-1034.1 19323220

[gcb16360-bib-0065] Shipley, B. (2013). The AIC model selection method applied to path analytic models compared using a d‐separation test. Ecology, 94(3), 560–564. 10.1890/12-0976.1 23687881

[gcb16360-bib-0066] Strauss, S. Y. , Webb, C. O. , & Salamin, N. (2006). Exotic taxa less related to native species are more invasive. Proceedings of the National Academy of Sciences of the United States of America, 103(15), 5841–5845. 10.1073/pnas.0508073103 16581902 PMC1421337

[gcb16360-bib-0067] Su, G. , Tedesco, P. A. , Toussaint, A. , Villéger, S. , & Brosse, S. (2022). Contemporary environment and historical legacy explain functional diversity of freshwater fishes in the world rivers. Global Ecology and Biogeography, 31(4), 700–713. 10.1111/geb.13455

[gcb16360-bib-0068] Swenson, N. G. (2014). Functional and phylogenetic ecology in R. Springer.

[gcb16360-bib-0069] Tan, J. , Pu, Z. , Ryberg, W. A. , & Jiang, L. (2015). Resident‐invader phylogenetic relatedness, not resident phylogenetic diversity, controls community invasibility. The American Naturalist, 186(1), 59–71. 10.1086/681584 26098339

[gcb16360-bib-0070] Thuiller, W. , Gallien, L. , Boulangeat, I. , De Bello, F. , Münkemüller, T. , Roquet, C. , & Lavergne, S. (2010). Resolving Darwin's naturalization conundrum: A quest for evidence. Diversity and Distributions, 16(3), 461–475. 10.1111/j.1472-4642.2010.00645.x

[gcb16360-bib-0071] Toussaint, A. , Charpin, N. , Beauchard, O. , Grenouillet, G. , Oberdorff, T. , Tedesco, P. A. , Brosse, S. , & Villéger, S. (2018). Non‐native species led to marked shifts in functional diversity of the world freshwater fish faunas. Ecology Letters, 21(11), 1649–1659. 10.1111/ele.13141 30187690

[gcb16360-bib-0072] Villéger, S. , Brosse, S. , Mouchet, M. , Mouillot, D. , & Vanni, M. J. (2017). Functional ecology of fish: Current approaches and future challenges. Aquatic Sciences, 79(4), 783–801. 10.1007/s00027-017-0546-z

[gcb16360-bib-0073] Watanabe, S. , & Opper, M. (2010). Asymptotic equivalence of Bayes cross validation and widely applicable information criterion in singular learning theory. Journal of Machine Learning Research, 11(12), 3571–3594.

[gcb16360-bib-0074] Webb, C. O. , Ackerly, D. D. , Mcpeek, M. A. , & Donoghue, M. J. (2002). Phylogenies and community ecology. Annual Review of Ecology and Systematics, 33(1), 475–505. 10.1146/annurev.ecolsys.33.010802.150448

[gcb16360-bib-0075] Yannelli, F. , Koch, C. , Jeschke, J. , & Kollmann, J. (2017). Limiting similarity and Darwin's naturalization hypothesis: Understanding the drivers of biotic resistance against invasive plant species. Oecologia, 183(3), 775–784. 10.1007/s00442-016-3798-8 28044207

[gcb16360-bib-0076] Zheng, Y. L. , Burns, J. H. , Liao, Z. Y. , Li, Y. P. , Yang, J. , Chen, Y. J. , Zhang, J. L. , & Zheng, Y. G. (2018). Species composition, functional and phylogenetic distances correlate with success of invasive Chromolaena odorata in an experimental test. Ecology Letters, 21(8), 1211–1220. 10.1111/ele.13090 29808558

