# Supplementary material for: Exotic fishes that are phylogenetically close but functionally distant to native fishes are more likely to establish
Source: Glob Chang Biol. 2022 Aug 4;28(19):5683–94. doi: 10.1111/gcb.16360 (PMC9543100; doi:10.1111/gcb.16360)
Supplement: Supplementary file 1 — Data S1 [file GCB-28-5683-s002.pdf]

Exotic fishes phylogenetically close but functionally distant to native ones are more likely to establish

Supplementary information

Meng Xu *et al.*

Table S1. Parameter estimates for the Bayesian hierarchical model to explain fish establishment success using multiple standardized explanatory variables (subtracting the mean and dividing by the standard deviation). Bold values are significantly different from zero (based on 95% credible intervals).

Predictors	Mean	SD	0.025 quant	0.15 quant	0.5 quant	0.85 quant	0.975 quant
**Introduction-native MPD**	**-0.41**	**0.11**	**-0.64**	**-0.53**	**-0.41**	**-0.29**	**-0.19**
**Introduction-native MFD**	**0.33**	**0.17**	**0.00**	**0.16**	**0.33**	**0.51**	**0.67**
Species richness	0.10	0.12	-0.13	-0.02	0.10	0.22	0.33
Native MPD	-0.03	0.11	-0.25	-0.14	-0.03	0.08	0.18
Native MFD	-0.08	0.14	-0.36	-0.23	-0.08	0.07	0.20
Area	-0.30	0.18	-0.70	-0.49	-0.28	-0.11	0.01
Elevation	0.01	0.11	-0.21	-0.10	0.01	0.13	0.24
Latitude	0.19	0.11	-0.02	0.08	0.19	0.31	0.41

Table S2. Parameter estimates for averaging generalized linear mixed model to explain fish establishment success using standardized explanatory variables (subtracting the mean and dividing by the standard deviation). Bold values denote the significant effects (P < 0.05). The model average is conducted by selecting and averaging the best fitted mixed models (ΔAIC<2).

	Estimate	SE	Adjusted SE	z value	Pr (>|z|)
**Introduction-native MPD**	**-0.43**	**0.11**	**0.11**	**3.86**	**0.00**
**Introduction-native MFD**	**0.32**	**0.15**	**0.15**	**2.07**	**0.04**
Species richness	0.01	0.05	0.05	0.26	0.79
Native MPD	-0.01	0.04	0.04	0.22	0.83
Native MFD	-0.05	0.10	0.10	0.48	0.63
Area	-0.24	0.18	0.18	1.36	0.17
Latitude	0.12	0.12	0.12	1.03	0.30

Table S3. The comparison of Candidate structural equation modelings (SEMs)

Model	Explanation	*Fisher's C*	*P*	K	AIC	ΔAIC	AIC weight
SEM1	Initial model	119.38	0.00	45	209.38	110.77	0.00
SEM2	Initial model plus effects of elevation on both similarity	27.04	0.00	47	121.04	22.43	0.00
**SEM3**	**Initial model plus effects of elevation and latitude on both similarity**	**0.61**	**0.96**	**49**	**98.61**	**0.00**	**0.54**
SEM4	Initial model plus effects of elevation and latitude on both similarity, and effect of area on phylogenetic similarity	0.13	0.94	50	100.13	1.52	0.25
SEM5	Initial model plus effects of elevation and latitude on both similarity, and effect of area on functional similarity	0.48	0.79	50	100.48	1.87	0.21

Table S4. Parameter estimates and standardized coefficients for the best-supported SEM

Response	Predictor	Estimate	SE	DF	*P* Value	Std. Coef
**Introduction outcome**	**Introduction-native MPD**	**-3.270**	**0.955**	**965.000**	**0.001**	**-0.172**
**Introduction outcome**	**Introduction-native MFD**	**0.543**	**0.237**	**965.000**	**0.022**	**0.165**
Introduction outcome	Species richness	0.026	0.035	965.000	0.458	0.038
Introduction outcome	Native MPD	-0.211	0.988	965.000	0.831	-0.010
Introduction outcome	Native MFD	-0.139	0.164	965.000	0.396	-0.051
**Introduction outcome**	**Area**	**0.000**	**0.000**	**965.000**	**0.045**	**-0.134**
Introduction outcome	Elevation	0.000	0.001	965.000	0.937	0.004
Introduction outcome	Latitude	0.051	0.034	965.000	0.132	0.073
**Introduction-native MPD**	**Native MPD**	**0.371**	**0.031**	**856.602**	**0.000**	**0.332**
**Introduction-native MPD**	**Species richness**	**0.005**	**0.001**	**700.967**	**0.000**	**0.145**
**Introduction-native MPD**	**Native MFD**	**0.027**	**0.004**	**778.814**	**0.000**	**0.188**
**Introduction-native MPD**	**Elevation**	**0.000**	**0.000**	**726.806**	**0.000**	**-0.179**
**Introduction-native MPD**	**Latitude**	**-0.006**	**0.001**	**573.448**	**0.000**	**-0.154**
**Introduction-native MFD**	**Native MFD**	**0.428**	**0.016**	**763.396**	**0.000**	**0.518**
**Introduction-native MFD**	**Species richness**	**0.011**	**0.004**	**667.422**	**0.007**	**0.054**
Introduction-native MFD	Native MPD	0.093	0.122	846.275	0.449	0.014
Introduction-native MFD	Elevation	0.000	0.000	722.272	0.282	-0.024
Introduction-native MFD	Latitude	0.003	0.005	535.617	0.495	0.015
**Native MPD**	**Elevation**	**0.000**	**0.000**	**706.876**	**0.023**	**-0.105**
**Native MPD**	**Latitude**	**-0.005**	**0.002**	**715.127**	**0.001**	**-0.160**
**Native MPD**	**Area**	**0.000**	**0.000**	**658.347**	**0.028**	**-0.080**
**Native MFD**	**Elevation**	**-0.001**	**0.000**	**698.525**	**0.000**	**-0.186**
**Native MFD**	**Latitude**	**-0.080**	**0.012**	**709.917**	**0.000**	**-0.312**
Native MFD	Area	0.000	0.000	665.043	0.192	-0.046
**Species richness**	**Elevation**	**-0.002**	**0.000**	**965.000**	**0.000**	**-0.387**
**Species richness**	**Latitude**	**-0.031**	**0.006**	**965.000**	**0.000**	**-0.177**
**Species richness**	**Area**	**0.000**	**0.000**	**965.000**	**0.000**	**0.129**
**~~Introduction-native MPD**	**~~ Introduction-native MFD**	**0.285**	**NA**	**965.000**	**0.000**	**0.285**
**~~Native MPD**	**~~Species richness**	**-0.191**	**NA**	**965.000**	**0.000**	**-0.191**
**~~native MFD**	**~~Species richness**	**-0.053**	**NA**	**965.000**	**0.050**	**-0.053**
**~~Native MPD**	**~~native MFD**	**0.446**	**NA**	**965.000**	**0.000**	**0.446**

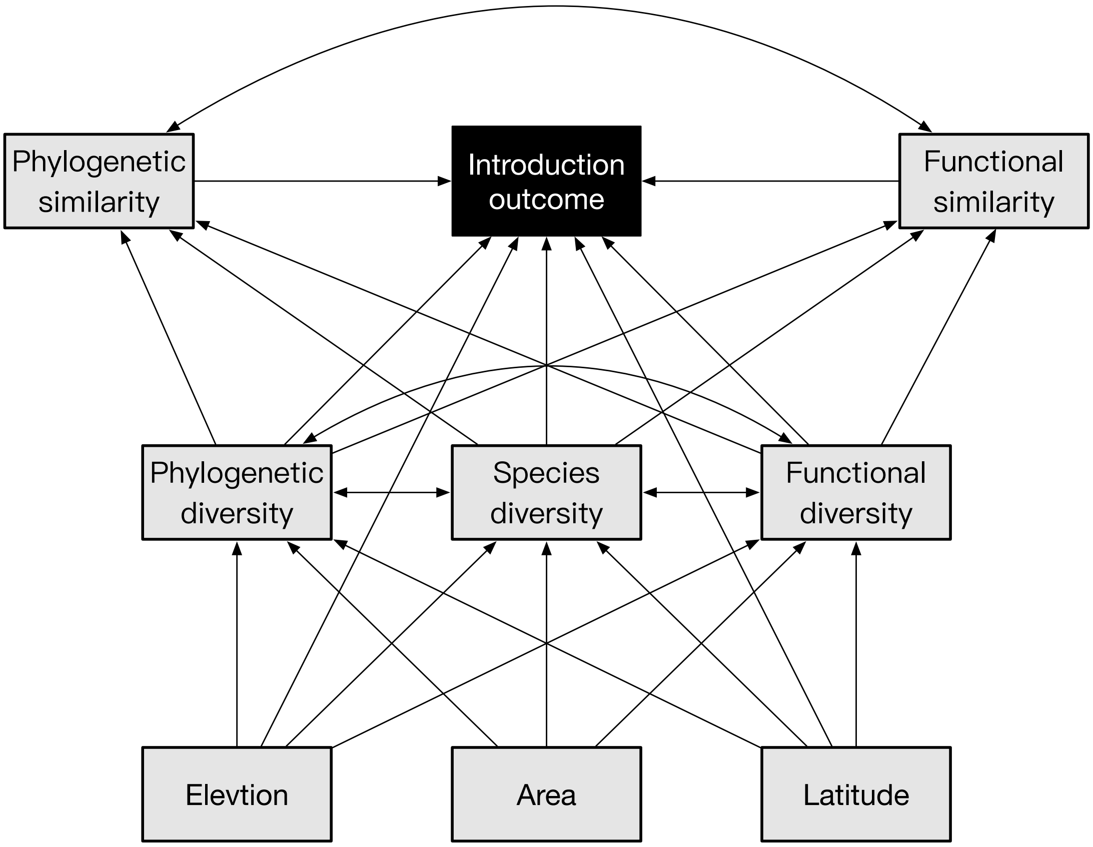

Figure S1. The initial model for structural equation modeling (SEM) exploring the effects of similarity, diversity, and geographical factors on the establishment of exotic fish species.

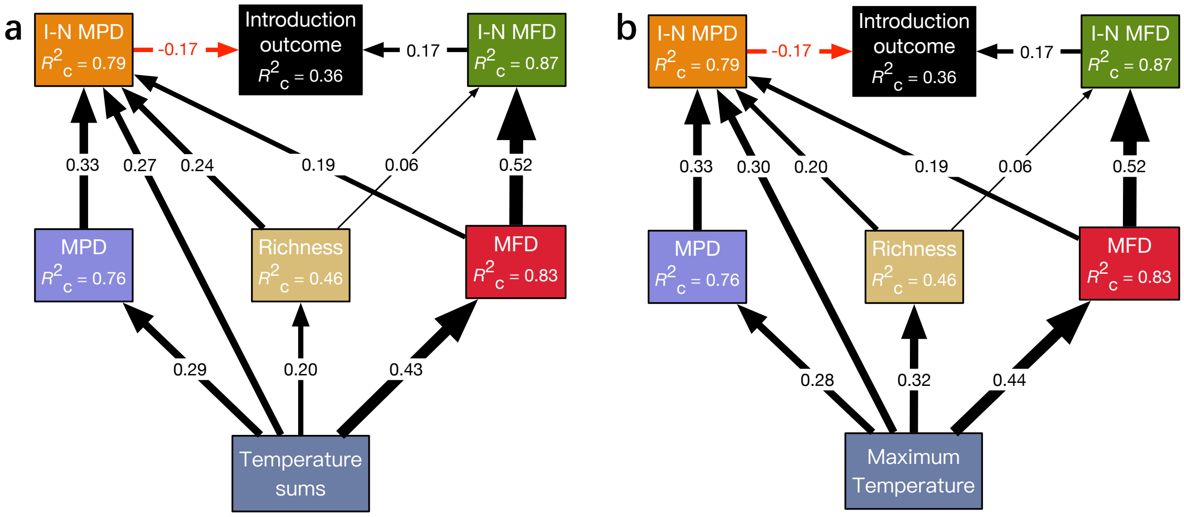

Figure S2. Structural equation modeling (SEM) exploring the effects of similarity, diversity on the establishment of exotic fish species considering the accumulated water temperature (a) and maximum water temperature (b). Both SEMs fit the data well (a: Fisher's $C = 0.083$, d.f. = 2, $P = 0.959$; $K = 38$, $n = 965$; b: Fisher's $C = 0.408$, d.f. = 2, $P = 0.816$; $K = 38$, $n = 965$). For clarity, only the significant paths ($P < 0.05$) are shown in the figures. Boxes represent measured variables and arrows represent relationships among variables. Black arrows denote positive relationships and red arrows negative ones. Standardized path coefficients are given for each significant path, the width of which is scaled by the magnitude of the standardized path coefficient. Conditional R_c^2 (based on both fixed and random effects) for each endogenous variable are reported in the corresponding boxes.
