# Supplementary material for: Exotic fishes that are phylogenetically close but functionally distant to native fishes are more likely to establish
Source: Glob Chang Biol. 2022 Aug 4;28(19):5683–94. doi: 10.1111/gcb.16360 (PMC9543100; doi:10.1111/gcb.16360)
Supplement: Supplementary file 2 — Data S2 [file GCB-28-5683-s001.pdf]

Exotic fishes phylogenetically close but functionally distant to native ones are more likely to establish

Supplementary information (section 2) for the nearest indexes

Meng Xu *et al.*

Table S1. Parameter estimates for the best Bayesian hierarchical model to explain fish establishment success using multiple standardized explanatory variables (subtracting the mean and dividing by the standard deviation). Bold values are significantly different from zero (based on 95% credible intervals).

Predictors	Mean	SD	0.025 quant	0.15 quant	0.5 quant	0.85 quant	0.975 quant
Introduction-native MNTD	-0.12	0.11	-0.35	-0.24	-0.12	-0.01	0.10
Introduction-native MNFD	-0.24	0.15	-0.54	-0.40	-0.24	-0.08	0.06
Species richness	0.03	0.14	-0.25	-0.12	0.03	0.18	0.31
Native MNTD	-0.10	0.13	-0.35	-0.23	-0.10	0.03	0.15
Native MNFD	0.14	0.12	-0.08	0.02	0.14	0.26	0.38
Area	-0.29	0.18	-0.68	-0.47	-0.27	-0.11	0.01
Elevation	0.09	0.11	-0.13	-0.03	0.09	0.20	0.31
**Latitude**	**0.27**	**0.11**	**0.06**	**0.16**	**0.27**	**0.39**	**0.49**

Table S2. Parameter estimates for averaging generalized linear mixed model to explain fish establishment success using standardized explanatory variables (subtracting the mean and dividing by the standard deviation). Bold values denote the significant effects (*P* < 0.05). The model average is conducted by selecting and averaging the best fitted mixed models (ΔAIC<2).

	Estimate	SE	Adjusted SE	z value	Pr (>|z|)
Introduction-native MNTD	-0.03	0.08	0.08	0.42	0.67
Introduction-native MNFD	-0.14	0.16	0.16	0.86	0.39
Species richness	0.00	0.03	0.03	0.05	0.96
Native MNTD	-0.01	0.04	0.04	0.20	0.84
Native MNFD	0.00	0.03	0.03	0.09	0.93
Area	-0.29	0.17	0.17	1.73	0.08
Elevation	0.01	0.04	0.04	0.17	0.86
**Latitude**	**0.28**	**0.09**	**0.09**	**2.99**	**0.00**

Table S3. Candidate structural equation modelings (SEMs)

Model	Explanation	*Fisher's C*	*P*	K	AIC	ΔAIC	AIC weight
SEM1	Initial model	87.49	0.00	45	177.49	77.45	0.00
SEM2	Initial model plus effects of elevation on both similarity	12.81	0.12	47	106.81	6.77	0.01
**SEM3**	**Initial model plus effects of elevation and latitude on both similarity**	**2.04**	**0.73**	**49**	**100.04**	**0.00**	**0.44**
SEM4	Initial model plus effects of elevation and latitude on both similarity, and effect of area on phylogenetic similarity	1.22	0.54	50	101.22	1.18	0.24
SEM5	Initial model plus effects of elevation and latitude on both similarity, and effect of area on functional similarity	0.82	0.66	50	100.82	0.78	0.30

Table S4. Parameter estimates and standardized coefficients for the best-supported model

Response	Predictor	Estimate	SE	DF	*P* Value	Std. Coef
Introduction outcome	Introduction-native MNTD	-0.546	0.538	965.000	0.310	-0.048
Introduction outcome	Introduction-native MNFD	-0.287	0.236	965.000	0.224	-0.076
Introduction outcome	Species richness	0.006	0.043	965.000	0.885	0.009
Introduction outcome	Native MNTD	-0.458	0.645	965.000	0.478	-0.038
Introduction outcome	Native MNFD	0.182	0.170	965.000	0.284	0.051
**Introduction outcome**	**Area**	**0.000**	**0.000**	**965.000**	**0.046**	**-0.126**
Introduction outcome	Elevation	0.001	0.001	965.000	0.501	0.031
**Introduction outcome**	**Latitude**	**0.077**	**0.033**	**965.000**	**0.019**	**0.105**
Introduction-native MNTD	Native MNTD	0.001	0.039	850.058	0.980	0.001
**Introduction-native**	**Species richness**	**-0.011**	**0.002**	**686.457**	**0.000**	**-0.177**
**Introduction-native**	**Native MNFD**	**0.077**	**0.009**	**847.717**	**0.000**	**0.246**
**Introduction-native**	**Elevation**	**0.000**	**0.000**	**725.412**	**0.000**	**-0.159**
Introduction-native MNTD	Latitude	-0.001	0.002	592.515	0.613	-0.017
**Introduction-native**	**Native MNFD**	**0.102**	**0.021**	**839.250**	**0.000**	**0.108**
**Introduction-native**	**Species richness**	**-0.026**	**0.005**	**626.991**	**0.000**	**-0.136**
Introduction-native MNFD	Native MNTD	0.055	0.086	821.442	0.521	0.017
**Introduction-native**	**Elevation**	**0.000**	**0.000**	**718.785**	**0.020**	**-0.056**
**Introduction-native**	**Latitude**	**-0.012**	**0.005**	**542.116**	**0.010**	**-0.062**
**Native MNTD**	**Elevation**	**0.000**	**0.000**	**703.053**	**0.000**	**0.170**
Native MNTD	Latitude	0.005	0.003	713.974	0.104	0.078
**Native MNTD**	**Area**	**0.000**	**0.000**	**662.359**	**0.000**	**-0.131**
Native MNFD	Elevation	0.000	0.000	702.870	0.259	0.054
Native MNFD	Latitude	-0.008	0.010	716.298	0.461	-0.037
**Native MNFD**	**Area**	**0.000**	**0.000**	**663.375**	**0.009**	**-0.099**
**Species richness**	**Elevation**	**-0.002**	**0.000**	**965.000**	**0.000**	**-0.387**
**Species richness**	**Latitude**	**-0.031**	**0.007**	**965.000**	**0.000**	**-0.177**
**Species richness**	**Area**	**0.000**	**0.000**	**965.000**	**0.000**	**0.129**
**~~Introduction-native**	**~~Introduction-native**	**0.278**	**NA**	**965.000**	**0.000**	**0.278**
**~~Native MNTD**	**~~Species richness**	**-0.534**	**NA**	**965.000**	**0.000**	**-0.534**
**~~native MNFD**	**~~Species richness**	**-0.392**	**NA**	**965.000**	**0.000**	**-0.392**
**~~Native MNTD**	**~~native MNFD**	**0.575**	**NA**	**965.000**	**0.000**	**0.575**

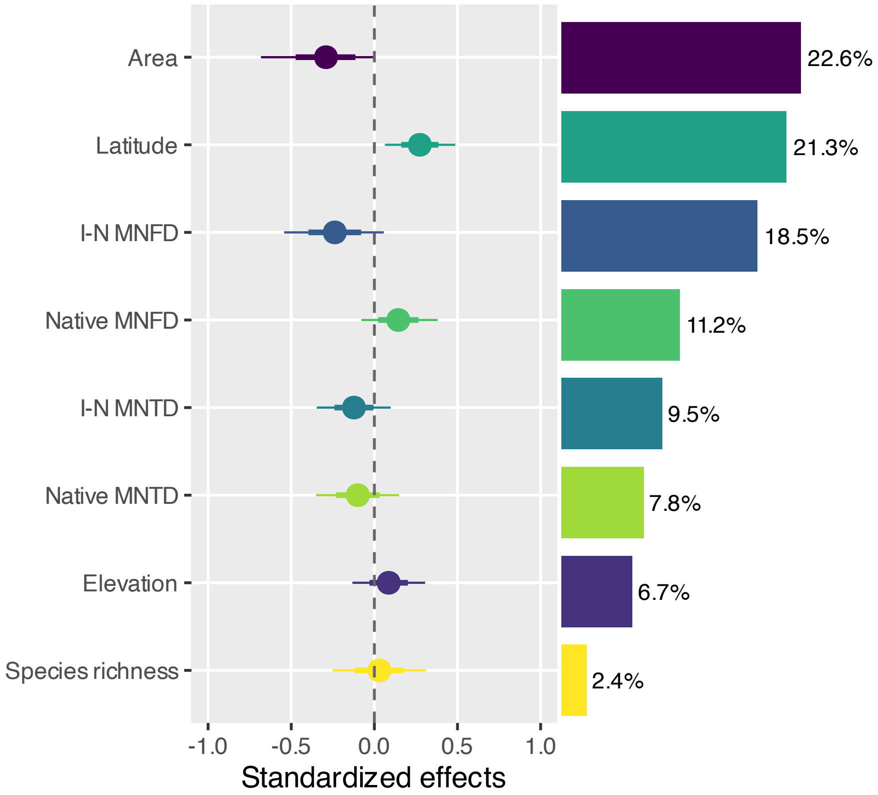

Figure S1. The effects and relative importance of predictive variables in explaining the establishment success of exotic fishes. The standardized effect size (± 95% and 70% credible intervals) was derived from Bayesian hierarchical model with predictive variables being standardized (by subtracting the mean and dividing by the standard deviation). The relative importance of each predictor, expressed as the percentage of explained variance, was calculated using the ratio between its standardized parameter estimate and the sum of all standardized parameter estimates. Dashed lines indicate effect = 0. Positive effects (the 95% credible interval does not include zero) indicate that the establishment probability increases with increasing values of predictive variables, while the negative effects indicate the opposite. MNTD denotes the mean nearest phylogenetic distance of native species, while MNFD denotes the mean nearest functional distance. I-N MNTD denotes the mean nearest phylogenetic distance between introduced exotic species and native species, while the I-N MNFD denotes the mean nearest functional distance between introduced exotic species and native species.

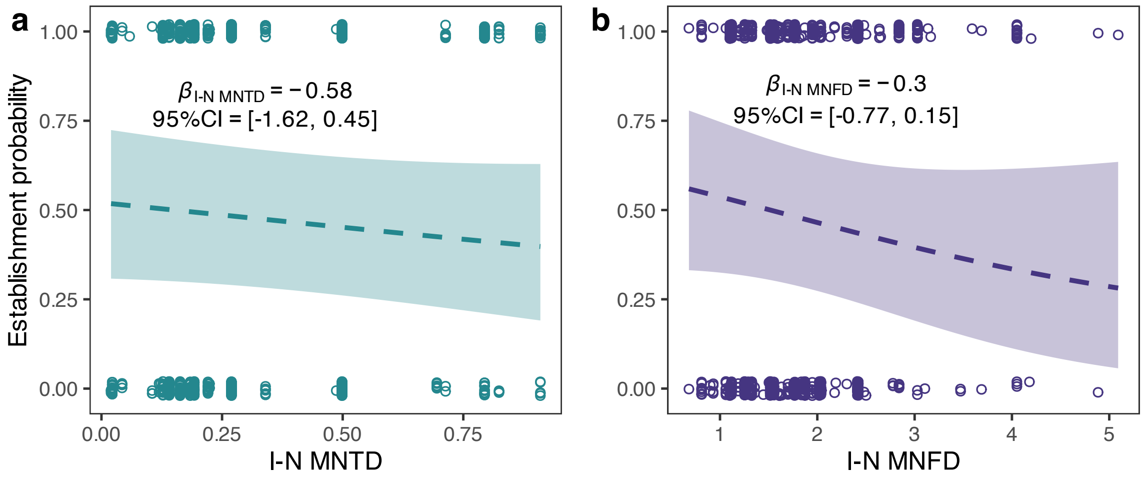

Figure S2. The respective relationships between the probability of successful establishment and introduced-native nearest phylogenetic distance (a), introduced-native nearest functional distance (b). The predictive curves (with 95% credible bands) were derived from the Bayesian hierarchical models with fixing the other predictive variables at the mean values. To make the relationships straightforward, the models were fitted with the raw values of predictive variables.

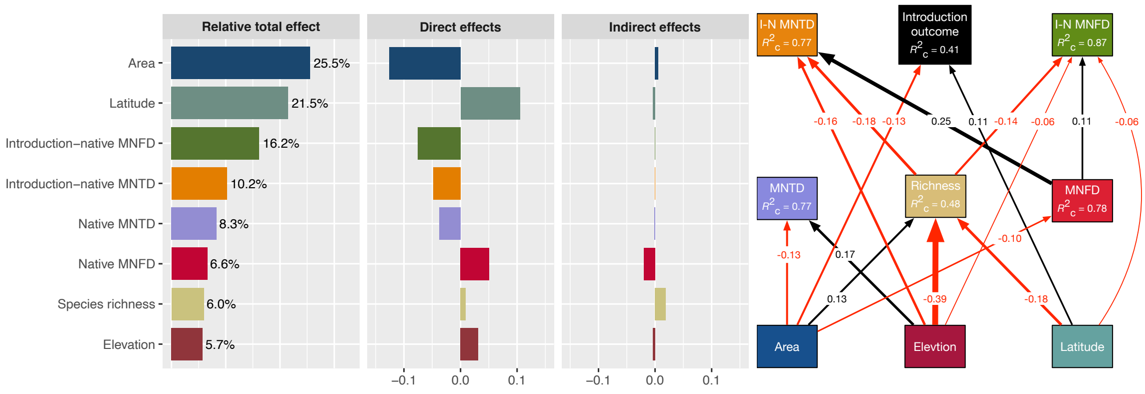

Figure S3. Structural equation modeling (SEM) exploring the effects of similarity, diversity, and geographical factors on the establishment of exotic fish species. MNTD denotes the mean nearest phylogenetic distance of native species, while MNFD denotes the mean nearest functional distance (*i.e.* phylogenetic and functional diversity respectively). I-N MNTD denotes the mean nearest phylogenetic distance between introduced exotic species and native species, while the I-N MNFD denotes the mean nearest functional distance between introduced exotic species and native species (*i.e.* exotic-native phylogenetic and functional similarity respectively). This SEM is determined through model comparison and fits the data well (Fisher's C = 2.042, d.f. = 4, P = 0.728; K = 49, n = 965) and, for clarity, only the significant paths (P < 0.05) are shown in the figure. Boxes represent measured variables and arrows represent relationships among variables. Black arrows denote positive relationships and red arrows negative ones. Standardized path coefficients are given for each significant path, the width of which is scaled by the magnitude of the standardized path coefficient. Conditional R_c^2 (based on both fixed and random effects) for each endogenous variable are reported in the corresponding boxes. The relative total effects, direct effects, and indirect effects on the establishment of exotic species are calculated and shown in the left part of this figure.
